# Improving patient experience for people prescribed medicines with a risk of dependence or withdrawal: co-designed solutions using experience based co-design

**DOI:** 10.1186/s12875-023-02253-9

**Published:** 2024-01-06

**Authors:** Jennifer Seddon, Claire Friedrich, Sarah Wadd, David Dicks, Sion Scott, Anthea Robinson, Charlotte Walker

**Affiliations:** 1 Centre for Psychological Research, Oxford Brookes University, Oxford, UK; 2 Department of Psychiatry, University of Oxford, Oxford, UK; 3 Substance Misuse and Ageing Research Team, University of Bedfordshire, Luton, UK; 4 School of Healthcare, University of Leicester, Leicester, UK; 5 East London NHS Foundation Trust, London, UK

**Keywords:** Experience based Co-design, Prescription medication dependence and withdrawal, Patient experience

## Abstract

**Background:**

Significant concerns have been raised regarding how medications with a risk of dependence or withdrawal are managed and how care is experienced by patients. This study sought to co-design solutions to improve the experience of care for patients prescribed benzodiazepines, z-drugs, opioids for chronic non-cancer pain, gabapentinoids and antidepressants.

**Method:**

Twenty patients and fifteen healthcare professionals from five different GP practices were recruited to take part. The study used Experience Based Co-Design. Patients and healthcare professionals completed semi-structured interviews and took part in feedback groups and co-design workshops to collaboratively identify priorities for improvement and to co-design solutions to improve the experience of care.

**Results:**

Poor patient experience was common among people prescribed medications with a risk of dependence or withdrawal. Patients and healthcare professionals identified three main priority areas to improve the experience of care: (i) ensuring patients are provided with detailed information in relation to their medication, (ii) ensuring continuity of care for patients, and (iii) providing alternative treatment options to medication. Solutions to improve care were co-designed by patients and healthcare staff and implemented within participating GP practices to improve the experience of care.

**Conclusion:**

Good patient experience is a key element of quality care. This study highlights that the provision of in-depth medication related information, continuity of care and alternative treatment to medication are important to patients prescribed medicines with a risk of dependence or withdrawal. Improving these aspects of care should be a priority for future improvement and delivery plans.

**Supplementary Information:**

The online version contains supplementary material available at 10.1186/s12875-023-02253-9.

## Background

Public Health England [1], the British Medical Association [2] and others [3–5] have identified poor management and poor patient experience in relation to the prescription, management and deprescription of medications that have a risk of dependence or withdrawal. In the United Kingdom (UK), these medications are widely used, with the scale of prescribing identified as a significant public health issue [1]. A recent report by Public Health England found 26% of adults in England, equivalent to 11.5 million people, were prescribed medications with a risk of dependence or withdrawal in a one-year period. These medications included benzodiazepines, z-drugs, gabapentinoids, opioids and antidepressants^1^ [1].

Rates of use and increases in prescribing similar to the UK have been documented in the US, Australia, Canada and several European countries for antidepressants, benzodiazepines and gabapentiniods [1, 3, 6–10]. Medicines with a risk of dependence or withdrawal such as benzodiazepines, opioids for chronic pain, and z-drugs should only be prescribed for a limited period [11–14], but many patients exceed the recommended duration of use, increasing the risk of dependence [1]. Dependence and withdrawal can mean that people fail in their attempts to stop taking medication resulting in medication being taken for longer or in higher doses than is safe or clinically appropriate.

Evidence suggests that many patients are not aware these medications have the potential to cause dependence or withdrawal, with inadequate information provided to patients [2, 4, 15]. The absence of information means patients are unable to make an informed choice as to whether medication is the right form of treatment for them, and are unprepared for any side effects or withdrawal symptoms that may arise. Concerns in relation to adverse effects, including tolerance and dependence, have led to patients using medication symptomatically or strategically, or by adjusting the dose to avoid unwanted side effects [16, 17]. Lack of support during the deprescription process has also been highlighted as a serious concern [2, 5], along with deprescription regimes that are poorly managed and result in significant withdrawal symptoms and patient distress [2, 4].

Good patient experience is a key element of quality care, alongside providing clinical excellence and safer care [18]. Patient experience is important not only because of its intrinsic value, but it is also justified on more utilitarian grounds as a means of improving patient safety and clinical effectiveness [19]. Understanding patient experience is necessary to guide service improvement [20].

Experience based co-design (EBCD) is an approach to improve healthcare services that brings together patients and healthcare staff to co-design service improvements based on lived experience. Involving patients in the design of healthcare services has long been regarded to result in services that are more patient-centred and more likely to address patients’ needs [21].

Using EBCD methods, this study aimed to improve the quality and experience of care for patients prescribed benzodiazepines, z-drugs, opioids for chronic non-cancer pain, gabapentinoids and antidepressants.

## Methods

### Setting and participant recruitment

Five GP practices in South East England were recruited to take part in the study, from which we aimed to recruit 15 healthcare professionals and 20 patients. To be eligible to take part patients needed to be aged 18+, have taken benzodiazepines, z-drugs, opioids for chronic non-cancer pain, antidepressants or gabapentinoids for a period of at least 12 months and to have stopped taking medication within the last 12 months.

Recruitment was via a computer search of practices’ records to identify patients that met study inclusion criteria, after which random selection was used to select patients to be invited to take part in the study. Patients were invited to participate via a mail-out sent by the GP practice. The recruitment procedure was amended part way through the project as the Covid-19 pandemic resulted in staff shortages making patient recruitment difficult. The amended method of recruitment involved clinicians identifying eligible patients; patients were then contacted by letter and invited to take part in the study. Patients who expressed an interest in participating were contacted by the research team. To ensure there was a diversity of experience in the quality of care received patients were asked to rate their experience of care from very negative to very positive.

Healthcare professionals were invited to take part by the practice manager. To be eligible, healthcare staff needed to play a key role in the prescription, management or deprescription of medicines.

### Study design

The study used a form of participatory action research known as Experience Based Co-Design (EBCD). EBCD involves patients and healthcare staff working collaboratively to identify areas for service improvement and to co-design solutions to improve the experience of care.

The first stage in the EBCD process involved conducting filmed or audio recorded semi-structured interviews with patients. These were held online or by phone and lasted 1–2 h. Interviews explored patients’ experiences of care including being prescribed medication, medication management and the process of deprescription. Semi-structured interviews with healthcare professionals focused on the experience of providing care to people prescribed medication with a risk of dependence or withdrawal (see supplementary files for interview schedules). Interviews lasted up to one hour, these were held online or by phone and audio recorded.

Interviews were transcribed verbatim. Codebook Thematic Analysis [22] using a framework approach [23] was used to analyse data. Coding frames were developed based on analysis of a sub-set of interview transcripts, along with input from the project PPI group. Separate coding frames were developed for patient and healthcare staff interviews.

Filmed interview footage was reviewed to identify the most salient aspects of the patient experience and edited to create a 30 min film divided into phases of the patient journey (i.e. starting, taking and stopping medication).

During the second stage of the project, patients and healthcare professionals took part in three feedback events and three co-design workshops. All events were held online to maximise attendance. Findings from interviews and the patient film were presented at feedback groups, with group discussion to trigger further dialogue and to identify and agree key priorities for improvement. Participants voted for the priorities to take forward to the co-design workshops. At each of the co-design workshops patients and healthcare staff worked collaboratively to identify solutions to improve the experience of care for each priority area. Feedback and co-design events were well attended by patients (mean attendance 7.4 patients per session, range 5–9 patients) and healthcare professionals (mean attendance: 4.4 staff members per session, range 3–6 staff). An evaluation event was held at the end of the study to map changes to service provision and patient experience.

## Results

### Participants

Fifteen healthcare professionals took part in the study (GPs n = 9, pharmacists n = 5, practice nurse n = 1). Length of experience ranged from 9 months to 23 years (mean: 5.9 years, SD: 6.3).

Twenty patients took part in the study. The majority of patients identified as female (female: n = 14, male: n = 6), the mean age of participants was 51 years (SD: 17.8, range 21–79 years). All patients were white British (data missing for four patients). Medication type included antidepressants (n = 5), gabapentinoids (n = 5), opioids (n = 4), benzodiazepines (n = 3) and z-drugs (n = 3). Length of medication usage ranged from 12 months to 40 years (mean: 6.8 years, SD: 10.2 years).

### Priorities for improvement

Despite recruiting people on the basis of both positive and negative experience of care, overall patient experience of care was poor. Poor experience of care included lack of patient information and medication reviews, and feeling unsupported during the process of deprescription. Positive patient experience included working in partnership with the clinical team and being involved in key decisions in relation to healthcare.

Three main areas were identified as priorities for improvement: (i) improved information and communication, (ii) continuity of care: the importance of seeing the same healthcare professional, and (iii) alternatives to medication and alternative ways to manage symptoms. Co-designed solutions for each of these priority areas are shown in Table 1.

#### Improved information and communication

Patients often felt they hadn’t been given enough information about the medication when it was first prescribed. Although patients were usually given information regarding dosage and some were told about potential side effects, patients were rarely given more in-depth information on the expected duration of prescription or how the medication would be managed. The limited information provided meant many patients did not realise the medication could result in dependence.

*Knowing more about what you’re going to take and how long you may have to stay on it and what effects it may have, I think would be useful to know from the start. It very often doesn’t get, it certainly doesn’t get discussed automatically. – (Patient, male, benzodiazepine)*.

Healthcare professionals recognised the importance of providing information, and many felt that adequate information was provided to patients at the point of prescription. However, the reason for prescription and the time available to have an in-depth discussion could limit how much information was given.

*If you’ve already spent quite a lot of time talking about other things, and then this comes up right at the end, you might be inclined to just prescribe it and say “oh try this and see how you get on” without much counselling, which I think probably happens quite a lot. – (General Practice based Pharmacist, female)*.

Patients felt it was important for detailed information to be provided at key stages of the patient pathway (i.e. at prescription, during medication reviews and at deprescription). Co-designed solutions to improve the experience of care are shown in Table 1.

#### Continuity of care: the importance of seeing the same healthcare professional

Collaboration between the patient and clinician was a valued aspect of care. Shared decision making was a key component of working in partnership; patients wanted to feel they had been listened to and that they had a voice in their how their care was managed. Trust, rapport and an established relationship with the clinician was central to this. However, many patients reported an absence of continuity in care, preventing the development of collaborative relationships and shared decision making. Patients described barriers to continuity of care such as appointment booking systems that prevented them from seeing the same clinician, or long waiting times if they wanted to see the same clinician.

*In an ideal world we’d all have a doctor that we could go and see who knew us, who’d got unlimited time to make sure that whatever you’re being prescribed and however they’re treating you suits you as an individual. But we’re not. Whether it’s going on the tablets or whether it’s coming off them I think you’re just put through a sausage machine a little bit. – (Patient, female, antidepressant)*.

Clinicians similarly felt continuity of care was important for managing medications safely and effectively and was especially important in the context of polypharmacy. Continuity of care enabled changes in symptoms to be identified more easily and the impact of treatment to be evaluated more effectively. Some of the participating GP practices had systems in place that enabled them to deliver continuity of care, but for the majority of GP practices, providing continuity of care was not possible.

*So, we have good continuity of care, so our morning appointments are with our registered patients and in the afternoon it’s people can come and see any doctor. So, there’s the flexibility that a patient can see another doctor, but if there’s a prescribing issue, so say for instance there’s some overuse of opioids, then there’s always a doctor who’s aware of the patient and can follow it through. I think it benefits patients and it benefits us, so that we prescribe safely really. – (GP, female)*.

Continuity of care during the process of deprescription was considered to be especially important as patients often felt they needed additional support in order to successfully reduce and stop using medication. General Practice based pharmacists were identified as being ideally suited to provide more regular support to patients undertaking deprescription; pharmacists have the pharmacological expertise to lead a reduction along with a greater degree of flexibility than GPs in offering more regular and extended appointments to patients. Co-designed solutions are shown in Table 1.

#### Alternatives to medication and alternative ways to manage symptoms

Patients reported that medication was typically offered as the first and sometimes only treatment option, with little or no information on alternative treatment options. Clinicians discussed the difficulty in accessing other forms of support, with services such as pain management and mental health support often having long waiting lists or limited provision. Alternative treatment options were often not discussed with patients due to the lack of availability of such services. Self-help techniques such as mindfulness and sleep hygiene practices were perceived as useful, but it was felt these approaches may not be suitable for all patients.

*I wish I’d had someone go “there’s this group that you could talk to about your condition, and they will talk through with you, you could try this or you could try this”. I’d like to have maybe had a doctor who would say “well actually [name], we don’t want to put you on medication, we want to do this”. – (Patient, female, gabapentinoid)*.

*I think if we could say, “You’re going to be seen within a month or two by a specialist dealing with pain and you’re going to get your psychological seminars and all of that, or you’re going to get your mental health,” if we’re talking about benzodiazepines for mental health and things, “You’re going to get your mental health therapy within a month or two, and it’s going to get started, and that’s all going to happen,” it would be a different conversation. But what we’re often seeing is that the wait times of those are getting longer and longer and sparser and sparser, so patients, it’s not fair to leave somebody six months or a year without offering them something that could help them potentially. – (GP, male)*.

Co-designing solutions for this action point was challenging, as ultimately patients and healthcare staff wanted better provision and availability of alternative treatment services, something that was beyond the scope of this study to address. Therefore, solutions instead focused on utilising local level provision where available, co-designed solutions are shown in Table 1.

**Table 1:** Priority areas and co-designed solutions to improve the experience of care

Priority area	Co-designed solutions
Improved information and communication	• Providing patients with easy to read, accessible information by signposting to available websites (e.g. www.patient.co.uk) • Conducting a ‘mini-review’ shortly after prescription • Ensuring patients are seen in-person for the prescribing appointment • Standardisation in the information provided within the GP practice • Medication related information provided to patients via text message following prescribing appointment • Group consultations to provide information and education to patients in relation to medication
Continuity of care: the importance of seeing the same healthcare professional	• Signposting patients to General Practice based pharmacists (if available) to provide consistency in support with deprescription • Where pharmacist support is limited, small groups of 2–3 GPs to work together to provide continuity of care • Ensuring medication reviews are conducted by the same clinician.
Alternatives to medication and alternative ways to manage symptoms	• Utilising services such as mental health link workers and social prescribing schemes where available • Providing information on all possible treatment options with patients, even if alternative treatment options and services are not available • Discussion of self-care treatment options

### Improvements to care

Changes to service provision as a result of the study included providing more information to patients at the point of prescription. For example, signposting patients to lay-friendly websites for additional medication related information, and sending patients an automated text message with additional information and links to further sources of support. Practices also reported offering extended appointments for patients during the process of deprescription to help ensure patients feel supported. Continuity of care was prioritised for patients undertaking deprescription, with work underway to try and ensure continuity of care during other stages of the patient pathway. One GP practice reported the creation of a specialised deprescription clinic; the results of this study informed the approach to care within the clinic. These changes were perceived to have improved the patient experience of care.

## Discussion

This study highlights the most salient priorities identified by patients and healthcare professionals to improve the experience of care for people prescribed medication with a risk of dependence or withdrawal. Three main areas were identified, these were: ensuring patients are provided with detailed information in relation to their medication, ensuring continuity of care, and providing alternative treatment options to medication. Co-designed solutions were identified for each of these areas to improve the experience of care within general practice.

Patients in this study reported not being given enough information about the medication they were prescribed. For some of the patients in this study, the lack of information meant they were unaware of the potential for dependence or withdrawal and were unprepared for any side effects that arose with continued use. These findings echo similar observations following calls for evidence by the British Medical Association [2] and the UK All Party Parliamentary Group for Prescribed Drug Dependence [4].

It is important that all patients are provided with adequate information in order to understand the potential risks and benefits of medication; this becomes increasingly important when the medication has the potential to result in adverse effects such as dependence or withdrawal. One of the key recommendations made by Public Health England [1] was to improve the information provided to patients and carers on prescribed medicines that had a risk of dependence or withdrawal. Involving patients in healthcare is a key requirement of the UK’s National Health Service (NHS) statutory duties [24], to do this effectively patients need to understand the potential risks and benefits of medication. Patients who are actively involved in decisions about their healthcare report greater satisfaction with their care and the decisions made in relation to their treatment [25].

In this study patients wanted both written accessible information as well as information provided in-person by the clinician. Clinicians were aware of the importance of providing information but acknowledged this can be challenging to do well in short appointment times, especially for patients with complex health conditions or polypharmacy. Only 8% of GPs feel that the standard consultation is long enough [26]. To compensate for the paucity of information provided, calls have been made for the creation of a dedicated website and helpline to provide detailed accessible information to patients prescribed medications with a risk of dependence or withdrawal [1]. This study suggests that the creation of a website or helpline would be a welcome addition to the available resources and would help to address the knowledge gap some patients have in relation to their medication. However, this should not be a substitute for information provided by clinicians at the point of prescription.

The co-designed solutions identified in this study offer practical ways to improve the information provided to patients, but clinicians need additional time to work with patients prescribed medication with a risk of dependence or withdrawal. Longer GP-patient consultations would give clinicians time to discuss the risks and benefits of medication in-depth, enabling patients to make an informed choice about their treatment; whilst more frequent appointments would provide opportunities to work collaboratively, review medication and agree next steps in the treatment plan. Although this may not be possible for all patient groups, prioritising longer and more frequent appointments for patients prescribed medicines with a risk of dependence or withdrawal may be beneficial.

Continuity of care has been described as an essential feature of general practice in the UK [27], with the Royal College of General Practitioners [28] noting the importance of a continuing GP-patient relationship. Continuity of care was highly valued but was not achieved for the majority of patients. Patients felt this negatively impacted on their experience of care and resulted in less opportunity to work in partnership with their healthcare provider.

Continuity of care has been associated with better healthcare outcomes and higher levels of patient satisfaction [29–31], but has become increasingly difficult to achieve in the UK as demand for healthcare in general practice increases and access to healthcare is prioritised [27, 32]. The proportion of patients who were able to see their preferred GP in 2022 fell to just 38% [33]. The national shortage of GPs has compounded the difficulty in realising continuity of care [34].

Several co-designed solutions were identified in this study to improve continuity of care. One of these was the creation of small clinical teams comprising 2–3 GPs or pharmacists who could provide continuity of care to patients. Relationship continuity is not necessarily restricted to just one clinician; patients value relationship continuity with several different clinicians, including GPs, pharmacists and practice nurses [27], and a ‘team-based’ approach to providing care is becoming increasingly recognised as important in the NHS [32]. The creation of ‘micro-teams’, whereby a small number of health professionals work together in collaboration, has been suggested previously as a way to achieve continuity of care. However the concept of micro-teams is still in its infancy and work is needed to evaluate their impact [34].

The decline in continuity of care has been identified as one of the most concerning impacts of the pressure on general practice, with relationship based care essential for patient safety and patient experience [32]. This study suggests that continuity of care is especially important for patients prescribed medicines with a risk of dependence or withdrawal and should be a priority when conducting medication reviews and during the process of deprescription.

The third area identified as being important for improving patient experience was the provision of alternative treatment options. The limited time available in GP-patient consultations is a barrier to alternative interventions [35], and very little consultation time is given to the discussion of treatment choice [36]. Clinicians may also find it difficult to identify viable alternative options for treatment, especially in in terms of psychotherapy [37]. Clinicians in this study felt they had a clinical responsibility to help patients, and similar to previous research, in situations where alternative treatments are limited, initiating a prescription was perceived to be justified [35, 38].

Patient choice and autonomy is an important aspect of healthcare. Research indicates that patients tend to accept the treatment recommendations of the clinician [39], meaning it is important to explore and discuss a range of treatment options. In cases where alternative treatments are not available or have extensive waiting lists, communicating why medication is being prescribed is important. Information in relation to how the medication will be managed and stopped should also be communicated.

Access to alternative care services is a recognised problem. NHS waiting times have steadily increased and have been exacerbated by the Covid-19 pandemic [40]. Public Health England [1] have called for improved information on non-pharmacological treatment options for patients. However, without timely access to alternative services, and with little available time within GP-patient consultation, prescription medication is likely to continue to be viewed as a first-line option for a range of health conditions.

Whilst some research has sought to explore the experiences of patients prescribed medicines with a risk of dependence or withdrawal, to our knowledge no research has positioned patients and healthcare professionals as active partners in identifying areas for change and in designing solutions to improve care. How medications with a risk of dependence or withdrawal are prescribed and managed, and how patients prescribed these medications experience care has been identified as a priority for both research and policy [1]. This study furthers our understanding of the patient experience of care, and presents feasible solutions to improve patient experience. In addition, most EBCD studies have been implemented within a single setting, this study demonstrates that EBCD methodology can be successfully used across multiple sites to bring about more wide-scale change.

However, the study does have some limitations. The GP practices taking part in this study were self-selected and so may not be representative of all GP practices. Recruitment of patients was done by clinical staff within each of the GP practices which may have resulted in an element of selection bias. However, the process of asking patients to rate their experience of care prior to participation resulted in a diverse sample, minimising impact of any potential selection bias. All patients in the study were white British. Given the ethnic disparities in access to healthcare and healthcare outcomes [41], future research should seek to understand the experiences of care of people from minority ethnic backgrounds who are prescribed these medications. A final limitation is the lack of formal evaluation within EBCD methodology to explore the longer-term impact of quality improvement processes. Although this study involved some evaluation with participating GP practices upon completion of the study, this could be expanded. Future studies should consider embedding a formal evaluation into the EBCD process.

The three priority areas identified in this study accord with recently published guidance by the National Institute for Health and Care Excellence (NICE) [42] and the NHS framework for action to optimise personalised care for people prescribed medicines with a risk of dependence or withdrawal [43]. These guidelines [42, 43] echo the importance of providing information and support to people when starting or stopping medications, as well as the importance of creating referral pathways to alternative care and treatment.

The results of this study highlight that much can be done to improve the quality and experience of care for people prescribed medications associated with dependence or withdrawal. Providing detailed information, continuity of care and alternative treatment to medication are highly valued by patients prescribed medicines with a risk of dependence or withdrawal. These aspects of care should be considered when commissioning services and developing local policies and need to be prioritised in improvement and delivery plans to improve the quality and experience of care.

### Electronic supplementary material

Below is the link to the electronic supplementary material.

Supplementary Material 1

## Data Availability

Due to ethical concerns, data cannot be made openly available in a repository.

## Footnotes

1 Antidepressants are not considered a dependence forming medication but do have a risk of withdrawal (National Institute for Health and Care Excellence, 2022).

## Abbreviations

EBCD: Experience-Based Co-Design
UK: United Kingdom
GP: General Practitioner
NHS: National Health Service
NICE: National Institute for Health and Care Excellence

## References

[1] Public Health England (2019). Dependence and withdrawal associated with some prescribed medicines: an evidence review.

[2] British Medical Association (2015). Prescribed Drugs associated with dependence and withdrawal – building a consensus for action.

[3] Cartagena Farias J, Porter L, McManus S, Strang J, Hickman M, Reed K (2017). Prescribing patterns in dependence forming medicines.

[4] Guy A, Brown M, Lewis S (2018). The patient Voice: an analysis of personal accounts of prescribed drug dependence and withdrawal submitted to petitions in Scotland and Wales.

[5] Read J, Gee A, Diggle J, Butler H (2019). Staying on, and coming off, antidepressants: the experiences of 752 UK adults. Addict Behav.

[6] Alabaku O, Yang A, Tharmarajah S, Suda K, Vigod S, Tadrous M (2023). Global trends in antidepressant, atypical antipsychotic, and benzodiazepine use: a cross-sectional analysis of 64 countries. PLoS ONE.

[7] Lembke A, Papac J, Humphreys K (2018). Our other prescription drug Problem. N Engl J Med.

[8] Pauly NJ, Delcher C, Slavova S, Lindahl E, Talbert J, Freeman PR (2020). Trends in gabapentin prescribing in a commercially insured US adult population, 2009–2016. J Managed care Specialty Pharm.

[9] Abbing-Karahagopian V, Huerta C, Souverein PC, de Abajo F, Leufkens HGM, Slattery J (2014). Antidepressant prescribing in five European countries: application of common definitions to assess the prevalence, clinical observations, and methodological implications. Eur J Clin Pharmacol.

[10] Mojtabai R, Olfson M (2013). National trends in long-term use of antidepressant medications: results from the US National Health and Nutrition Examination Survey. J Clin Psychiatry.

[11] Kennedy KM, O’Riordan J (2019). Prescribing benzodiazepines in general practice. Br J Gen Pract.

[12] Centers for Disease Control and Prevention (2016). Guideline for Prescribing opioids for Chronic Pain. J Pain Palliat Care Pharm.

[13] National Institute for Health and Care Excellence. Chronic pain (primary and secondary) in over 16s: assessment of all chronic pain and management of chronic primary pain. 2021. 33939353

[14] National Institute for Health and Care Excellence. What issues should I be aware of when prescribing Z-drugs? NICE, Clinical Knowledge Summaries;; 2022.

[15] Davies J, Pauli R, Montagu L, Lindsay R, Raymond-Barker B (2018). Antidepressant withdrawal: a survey of patients’ experience.

[16] Pound P, Britten N, Morgan M, Yardley L, Pope C, Daker-White G (2005). Resisting medicines: a synthesis of qualitative studies of medicine taking. Soc Sci Med.

[17] Britten N, Riley R, Morgan M (2010). Resisting psychotropic medicines: a synthesis of qualitative studies of medicine-taking. Adv Psychiatr Treat.

[18] NHS Institute for Innovation and Improvement. The patient experience book. https://www.england.nhs.uk/improvement-hub/wp-content/uploads/sites/44/2017/11/Patient-Experience-Guidance-and-Support.pdf; 2013.

[19] Berwick DM (2009). What ‘Patient-Centered’ should Mean: confessions of an Extremist. Health Aff.

[20] de Silva D (2013). Measuring patient experience.

[21] Coulter A, Locock L, Ziebland S, Calabrese J (2014). Collecting data on patient experience is not enough: they must be used to improve care. BMJ: Br Med J.

[22] Braun V, Clarke V (2022). Conceptual and design thinking for thematic analysis. Qualitative Psychol.

[23] Ritchie J, Spencer I, Bryman A, Burgess R (1994). Qualitative data analysis for applied policy research. Analyzing qualitative data.

[24] NHS England (2017). Involving people in their own health and care: statutory guidance for clinical commissioning groups and NHS England.

[25] Care Quality Commission (2016). Better care in my hands: a review of how people are involved in their care.

[26] British Medical Association (2015). National Survey of GPs. The future of general practice.

[27] Freeman G, Hughes J (2010). Continuity of care and the patient experience.

[28] Royal College of General Practitioners (2008). Good medical practice for General practitioners.

[29] Gray DJP, Sidaway-Lee K, White E, Thorne A, Evans PH (2018). Continuity of care with doctors—a matter of life and death? A systematic review of continuity of care and mortality. BMJ Open.

[30] Haggerty JL, Reid RJ, Freeman GK, Starfield BH, Adair CE, McKendry R (2003). Continuity of care: a multidisciplinary review. BMJ.

[31] Van Walraven C, Oake N, Jennings A, Forster AJ (2010). The association between continuity of care and outcomes: a systematic and critical review. J Eval Clin Pract.

[32] Health and Social Care Committee (2022). The future of general practice. Fourth report of session 2022-23.

[33] NHS England (2022). GP patient survey.

[34] Jeffers H, Baker M (2016). Continuity of care: still important in modern-day general practice. Br J Gen Pract.

[35] Sirdifield C, Anthierens S, Creupelandt H, Chipchase SY, Christiaens T, Siriwardena AN (2013). General practitioners’ experiences and perceptions of benzodiazepine prescribing: systematic review and meta-synthesis. BMC Fam Pract.

[36] Young HN, Bell RA, Epstein RM, Feldman MD, Kravitz RL (2008). Physicians’ Shared decision-making behaviors in Depression Care. Arch Intern Med.

[37] Wilson J, Read J (2001). What prevents GPs from using outside resources for women experiencing depression? A New Zealand study. Fam Pract.

[38] Anthierens S, Habraken H, Petrovic M, Christiaens T (2007). The lesser evil? Initiating a benzodiazepine prescription in general practice. Scand J Prim Health Care.

[39] Gibson K, Cartwright C, Read J (2014). Patient-centered perspectives on antidepressant use. Int J Mental Health.

[40] Pope C (2023). NHS waiting times: a government pledge. BMJ.

[41] Shavers VL, Klein WMP, Fagan P (2012). Research on Race/Ethnicity and Health Care discrimination: where we are and where we need to go. Am J Public Health.

[42] National Institute for Health and Care Excellence (2022). Medicines associated with dependence or withdrawal symptoms: safe prescribing and withdrawal management for adults.

[43] NHS England (2023). Optimising personalised care for adults prescribed medicines associated with dependence or withdrawal symptoms: Framework for action for integrated care boards (ICBs) and primary care.

