# Supplementary material for: Improving patient experience for people prescribed medicines with a risk of dependence or withdrawal: co-designed solutions using experience based co-design
Source: BMC Prim Care. 2024 Jan 6;25:17. doi: 10.1186/s12875-023-02253-9 (PMC10770999; doi:10.1186/s12875-023-02253-9)
Supplement: Supplementary file 1 — Supplementary Material 1 [file 12875_2023_2253_MOESM1_ESM.docx]

**INTERVIEW SCHEDULE – PATIENT VERSION**

Demographic questions:

- What is your age?
- What is your gender?
- What GP practice are you registered with?
- What prescription medications have you taken from the following: benzodiazepines, z-drugs (e.g. zopiclone, zolpidem, zaleplon), antidepressants, opioids (for chronic non-cancer pain), gabapentin or pregabalin?
- How long did you take this medication for?
- When did you stop taking this medication?
- Can you remember what dose of medication you used to take?

Interview questions:

- [Q1] Can you tell me about your experience of taking this medication?
- [Q2] Can you tell me about when you were first prescribed the medication?
  - Why were you prescribed it?
  - What information were you given about the medication?
  - How did you feel about taking this medication?
  - Do you feel you were given enough information at the time of initial prescription?
- [Q3] Can you tell me how the medication was managed when you were taking it?
  - Did you have regular medication reviews, if so, what did this involve? If not, why not?
  - Did you discuss on-going use of the medication with your GP? If yes, what information/topics did those conversations cover? If not, would you like to have had regular reviews of the medication with your GP, why?
  - Were you happy with how the medication was managed, and why?
- [Q4] Why did you stop taking the medication?
  - Was deprescription of the medication instigated by you or your healthcare professional? What was the reason for this?
- [Q5] How was the process of stopping this medication managed?
  - What were the steps involved in stopping the medication?
  - What role did your GP/ other health professionals play in this?
  - Did you use any others sources of support (e.g. online support groups/ forums)?
  - How did you contribute to the reduction plan?
  - How did you find the process of deprescription?
  - Were there any positive aspects to the process?
  - Were there any negative aspects to the process?
- [Q6] What do you feel was most important for you in shaping the overall experience of care?
- [Q7] What do you think are the major challenges or issues faced by people taking these medications?
- [Q8] What aspects of the care that you received were you happy or unhappy with?
- [Q9] What aspects of the care you received would you change, and why?
- [Q10] In your opinion, what could be improved for people prescribed medication with a risk of dependence or withdrawal?
- [Q11] How has the covid-19 pandemic affected the care you received? Did the pandemic result in any challenges or benefits for you in terms of your care?

**INTERVIEW SCHEDULE – HEALTHCARE PROFESSIONAL VERSION**

Demographic questions:

- What GP practice do you work in?
- What is your role in the practice?
- How long have you worked in this role?

Interview questions:

- [Q1] Could you tell me about your experience of prescribing medications with risks of dependence and/or withdrawal to patients?
  - What do you find works well for you in terms of prescribing these medications or providing care to people prescribed these medications?
  - What challenges have you experienced in prescribing these medications or in providing care to people prescribed these medications?
  - What do you find works well for patients in terms of prescribing these medications or supporting people prescribed these medications?
  - In your experience, what have patients prescribed these medications found challenging?
- [Q2] Can you tell me about your experience of deprescribing these medications to patients?
  - How do you typically manage the process of deprescription for these medications?
  - Can you talk me through the process step by step?
  - What do you find works well for you in terms of deprescribing these medications?
  - What have you noticed works well for patients in terms of deprescribing these medications?
  - How do you support patients going through the process of deprescription?
  - What are the challenges for you in deprescribing these medications?
  - Are you able to refer to other services if you feel it would be of benefit to patients?
- [Q3] What do you think are the major problems faced by patients when taking these medications?
- [Q4] What could be improved for patients prescribed this medication?
  - How could the experience be improved for healthcare professionals?
  - How could the experience be improved for patients?
- [Q5] What do you feel is done well in managing prescription medications in primary care, and in managing the process of deprescribing?
- [Q6] How do you meet this particular group of patients’ needs? Which needs do you feel are difficult to meet?
- [Q7] How could you be supported in working with patients prescribed these medications?
- [Q8] How has the covid-19 pandemic affected the care you are able to provide to patients? Has the pandemic resulted in any challenges? Have there been any benefits?
- [Q9] How do you feel the process of prescribing these medication and the process of deprescription could be improved?
